# Mesenteric Resistance Arteries in Type 2 Diabetic db/db Mice Undergo Outward Remodeling

**DOI:** 10.1371/journal.pone.0023337

**Published:** 2011-08-04

**Authors:** Flavia M. Souza-Smith, Paige S. Katz, Aaron J. Trask, James A. Stewart, Kevin C. Lord, Kurt J. Varner, Dalton V. Vassallo, Pamela A. Lucchesi

**Affiliations:** 1 Department of Pharmacology and Experimental Therapeutics, Louisiana State University Health Sciences Center, New Orleans, Louisiana, United States of America; 2 Department of Physiological Sciences, Federal University of Espirito Santo, Vitoria, Espirito Santo, Brazil; 3 Department of Physiology, Louisiana State University Health Sciences Center, New Orleans, Louisiana, United States of America; 4 Center for Cardiovascular and Pulmonary Research and The Heart Center, The Research Institute at Nationwide Children's Hospital, Columbus, Ohio, United States of America; 5 Department of Pediatrics, The Ohio State University, Columbus, Ohio, United States of America; 6 Feik School of Pharmacy, University of the Incarnate Word, San Antonio, Texas, United States of America; University of Arizona, United States of America

## Abstract

**Objective:**

Resistance vessel remodeling is controlled by myriad of hemodynamic and neurohormonal factors. This study characterized structural and molecular remodeling in mesenteric resistance arteries (MRAs) in diabetic (db/db) and control (Db/db) mice.

**Methods:**

Structural properties were assessed in isolated MRAs from 12 and 16 wk-old db/db and Db/db mice by pressure myography. Matrix regulatory proteins were measured by Western blot analysis. Mean arterial pressure and superior mesenteric blood flow were measured in 12 wk-old mice by telemetry and a Doppler flow nanoprobe, respectively.

**Results:**

Blood pressure was similar between groups. Lumen diameter and medial cross-sectional area were significantly increased in 16 wk-old db/db MRA compared to control, indicating outward hypertrophic remodeling. Moreover, wall stress and cross-sectional compliance were significantly larger in diabetic arteries. These remodeling indices were associated with increased expression of matrix regulatory proteins matrix metalloproteinase (MMP)-9, MMP-12, tissue inhibitors of matrix metalloproteinase (TIMP)-1, TIMP-2, and plasminogen activator inhibitor-1 (PAI-1) in db/db arteries. Finally, superior mesenteric artery blood flow was increased by 46% in 12 wk-old db/db mice, a finding that preceded mesenteric resistance artery remodeling.

**Conclusions:**

These data suggest that flow-induced hemodynamic changes may supersede the local neurohormonal and metabolic milieu to culminate in hypertrophic outward remodeling of type 2 DM mesenteric resistance arteries.

## Introduction

Diabetes mellitus (DM) is the fifth leading cause of death in the United States, and type 2 DM accounts for 90–95% of all diabetic cases for which vascular complications are a leading cause of morbidity, mortality and economic burden [1]. Chronic hyperglycemia, coupled with insulin resistance, is a hallmark of type 2 DM and underlies many of the observed pathophysiological complications in the cardiovascular system [2], [3]. Moreover, type 2 DM is associated with a cluster of risk factors including obesity, hypercholesterolemia, hyperlipidemia and hypertension. Together, these factors lead to increased accumulation of advanced glycation end products, alterations in the renin-angiotensin system (RAS), oxidative stress, and reduced nitric oxide bioavailability [4], [5], [6] culminating in endothelial dysfunction, altered vasoreactivity, and vessel wall remodeling [7], [8].

Although the regulation of resistance artery function has been extensively studied within the context of type 2 DM, much less is known about the mechanisms that dictate diabetes-associated structural remodeling of the microvasculature. Vascular remodeling involves the reorganization of existing cells and extracellular matrix (ECM) or changes in vascular smooth muscle cell (VSMC) growth and migration [9], and can be characterized as hypertrophic (increased cross-sectional area), eutrophic (no change in cross-sectional area) or hypotrophic remodeling (reduced cross-sectional area). These processes have been associated with either inward remodeling (smaller lumen diameter) or outward remodeling (increased lumen diameter) [10]. In small resistance arteries from type 2 diabetic patients, hypertrophic remodeling and reduced fibrosis has been reported [11]. In contrast, hypertensive patients with pre-existing type 2 DM had predominantly eutrophic remodeling and increased fibrosis [12].

Dynamic regulation of the ECM is an essential component of the remodeling process. The ECM not only dictates in part the integrity of the vessel wall, but is also an important determinant of vessel compliance; however, the molecular mechanisms that dictate ECM accumulation and turnover in diabetic microvessels have not been extensively studied. A balance between matrix metalloproteinases (MMPs) and tissue inhibitors of matrix metalloproteinases (TIMPs) is thought to play a central role in vessel remodeling, and an imbalance between MMPs and TIMPs may lead to excess ECM accumulation or degradation [13], [14]. For example, diabetic nephropathy and cardiomyopathy involves excessive ECM deposition due to a decrease in the MMP/TIMP ratio [15], [16]. The pro-fibrotic plasminogen activator inhibitor-1 (PAI-1) is increased in DM, insulin resistance, and hypertension [17].

In addition to neurohormonal regulation of ECM and VSMC growth, hemodynamics also dictate the pattern of microvascular remodeling. For example, outward remodeling occurs in response to chronic increases in blood flow in rat mesenteric resistance arteries [18]. Thus, the pattern of vascular remodeling in diabetes may be tissue-specific, reflecting a dynamic interaction between hyperglycemia, hemodynamic stimuli and locally generated angiotensin II, as well as oxidative stress and inflammation [19]. The aim of the present study was to examine mesenteric resistance arteriole (MRA) structure in leptin-receptor deficient db/db mice, a model of type 2 DM, insulin-resistance, hyperlipidemia and obesity.

## Materials and Methods

### Ethics Statement

This study conformed with the *Guide for the Care and Use of Laboratory Animals* published by the U.S. National Institutes of Health (NIH Publication No. 85–23, revised 1996) and was approved by the LSU Health Sciences Center Institutional Animal Care and Use Committee (IACUC protocol number 2498, Institutional Animal Assurance number A3094-01) and the Nationwide Children's Hospital Institutional Animal Care and Use Committee (IACUC protocol number AR08-00023, Institutional Animal Assurance Number A3544-01).

### Animals

All experiments used 12 and 16 wk-old male type 2 DM db/db (C57BL/KsJ-db-/db-) and control heterozygous Db/db mice (C57BL/KsJ-db+/db−, The Jackson Laboratory, Bar Harbor, ME, USA). By 4 wks of age db/db mice are obese, develop hyperglycemia by 8 wks of age, overt DM by 12 wks of age, and exhibit many common features of type 2 DM, including hyperlipidemia, obesity and insulin resistance. Heterozygous mice were used as control, which cannot be distinguished morphologically or physiologically from wild type mice (data not shown). Mice were housed under a 12-hour light/dark cycle at 22°C and 60% humidity. They were allowed *ad libitum* access to water and standard laboratory mouse chow.

### Blood Pressure and Blood Glucose Measurements

Radiotelemetry transmitter catheters were implanted into the right carotid arteries of 16 wk-old Db/db and db/db mice using aseptic surgical techniques in animals anesthetized with 2% isoflurane. A subcutaneous space was bluntly dissected in the right flank region to house the radiotelemetry transmitter (Model PhysioTel PA-C10; Data Sciences International, St. Paul, MN, USA). Baseline mean arterial blood pressure was recorded for 4 weeks in conscious, freely moving mice using Dataquest A.R.T (Data Sciences, St. Paul, MN, USA) acquisition software after the return of diurnal cycles (10 days post surgery). Following the acquisition of baseline blood pressure and heart rate, data were collected for one hour at the same time each day and averaged into 2-second bins over 7 days. Prior to the terminal vessel experiment and 12 hours after fasting, blood glucose was measured with an Accu-Chek II portable glucose meter (Roche, Indianapolis, IN, USA).

### Mesenteric Isolation

Db/db and db/db mice were anesthetized using ketamine (200 mg kg^−1^, ip) and xylazine (10 mg kg^−1^, ip). Mesenteric resistance arteries at the fourth to fifth branch of the superior mesenteric artery were dissected and isolated from surrounding adipose and connective tissue using a dissecting microscope and mounted onto two glass micropipettes in a video-monitored perfusion system as previously described [20]. The intravascular pressure was controlled by a pressure servo control (Living Systems Instrumentation, Burlington, VT, USA). Additional MRAs were isolated from separate sets of Db/db and db/db mice and either formalin fixed (10% phosphate buffered formalin) or frozen in liquid N_2_ for histological or biochemical experiments, respectively.

### Experimental Protocol

Isolated MRAs were bathed in a 4 mL chamber with physiological salt solution (PSS, pH 7.4, 37°C) containing (in mmol/L): 130 NaCl, 14.9 NaHCO_3_, 3.7 KCl, 1.6 CaCl_2_-2H_2_O, 1.2 KH_2_PO_4_, 1.2 MgSO_4_-7H_2_O, 11 glucose, and 10 HEPES. Arteries were equilibrated at 50 mmHg luminal pressure under no-flow conditions for 45 min and bathed in PSS at 37°C. Arteries were then maximally relaxed with Ca^2+^-free-PSS supplemented with 2 mmol/L EGTA and 100 µmol/L sodium nitroprusside. After 30 min equilibration, intraluminal pressure was recorded as the baseline passive diameter (PD). A passive pressure-diameter curve was generated by stepwise increases in intraluminal pressure from 10 to 125 mmHg. Both internal diameter (D_i_) and wall thickness (WT) were measured.

### Mesenteric Resistant Artery Measurements

The following structural and mechanical parameters were determined as previously described [20], [21]:

Media cross-sectional area (CSA)  = π (D_e_^2^−D_i_^2^) /4

Cross-sectional compliance (Ccs, µm^2^ mmHg^−1^)  = Δ*A*i/ΔP, in which Δ*A*i is the change in internal lumen cross-sectional area induced by a pressure change (ΔP).

Cross-sectional distensibility (Dcs, kPa^−1^) as Dcs = Ccs/*A*i

Circumferential wall strain (ε)  =  (D–D_0_) /D_0_, where D was the observed lumen diameter for a given intraluminal pressure and D_0_ was the original diameter measured at 10 mmHg intraluminal pressure.

Circumferential wall stress (σ)  =  (PD)/(2WT), where P is the intraluminal pressure and D is the lumen diameter. Pressure is converted to dynes per cm^2^ (1 mmHg = 1.3343×10^5^ dynes/cm^2^) when stress is examined as a function of strain.

Incremental Elastic Modulus (E_inc_)  = Δσ/Δε, which is used when the stress-strain curve is curvilinear rather than linear.

### Vascular Morphology

Formalin fixed, paraffin-embedded, fourth to fifth order isolated mesenteric resistance arteries from 16 wk-old mice were sectioned (6 µm), stained with picrosirius red (PSR) for collagen and quantified using picrosirius polarization methods [22]. The images were digitized using Olympus MicroSuite 5. Two sections per animal (n = 8) were used in each group. Data are expressed as collagen area percent and was normalized to medial cross sectional area.

### Western Blot Analysis

Tissue lysates from 16 wk-old mice were prepared from MRAs in a buffer containing 0.1% Triton X-100 and (in mmol/L); 50 NaCl, 1 MgCl_2_, 25 HEPES (pH 7.4), 2 EGTA, 10 NaF, 0.1 Na_3_VO_4_, 10 Na-Pyrophosphate, and 0.1% deoxycholate and protease inhibitors. Protein concentration was determined using the bicinchoninic acid assay (Pierce, Rockford, IL, USA). Equal amounts of protein from each sample (40–50 µg) were separated on 10% SDS-PAGE gels. Nitrocellulose membranes were stained with Ponceau Red to verify even transfer. Membranes were then blocked and incubated overnight at 4°C with the following primary antisera: anti-MMP-9, anti-TIMP-1, and anti-TIMP-2 (Millipore, formerly Chemicon, Collerica, MA, USA), anti-MMP-12 (Santa Cruz Biotechnology, Santa Cruz, CA, USA), anti-PAI-1 (BD Biosciences, formerly Transduction Laboratories, San Jose, CA, USA), anti-GAPDH (AbCam, Cambridge, MA, USA) as a loading control and horseradish peroxidase-conjugated secondary antisera (GE Healthcare, formerly Amersham Biosciences, Piscataway, NJ, USA). Blots were developed using SuperSignal reagents (Pierce, Rockford, IL, USA) and exposed on x-ray film. Immunoreactive bands were quantified by densitometry using an Alpha Innotech gel imaging system (Cell Biosciences, Santa Clara, CA, USA), and data were normalized to GAPDH levels.

### Blood Flow Measurements

Direct hemodynamic measures of superior mesenteric blood flow were performed in 12 wk-old male Db/db (n = 5) and db/db (n = 6) mice. Mice were maintained on isoflurane anesthesia (2.25%) at a body temperature of 37°C. The superior mesenteric artery was carefully isolated and a Doppler flow nanoprobe (Model 0.7PSB, Transonic Systems, Inc., Ithaca, NY) connected to a TS420 perivascular flowmeter (Transonic Systems, Inc., Ithaca, NY, USA) was gently placed around the artery. A 3-minute average of mesenteric blood flow was recorded and analyzed after a 25-minute equilibration period using LabScribe 2 data acquisition and analysis software (iWORX Systems, Inc., Dover, NH, USA).

### Data Analysis

Unpaired Student's t-test or two-way repeated measures ANOVA was used when appropriate. Data are presented as mean ± SEM, with *p*<0.05 representing significance.

## Results

### Blood Pressure and Blood Glucose

At 16 wks of age, there was no difference in systolic blood pressure between groups (Table 1). Diabetic db/db mice displayed significantly higher blood glucose concentrations and body weight compared to Db/db mice (Table 1).

**Table 1:** Physiological parameters of control and diabetic mice.

	Control (Db/db)	Diabetic (db/db)
**Blood Glucose (mg/dL)**	128±10	506±35***
**Body Weight (g)**	29.8±0.4	55.6±1***
**Systolic Blood Pressure (mmHg)**	116±2.7	114±1.7

Unpaired t-test: Db/db vs db/db,

***p<0.001, means ± SEM. Blood glucose and body weight, n = 10 for each group. Blood pressure, n = 6 for each group.

### Mesenteric Resistance Artery Structure in 16 wk-old Mice

**Figure 1:**
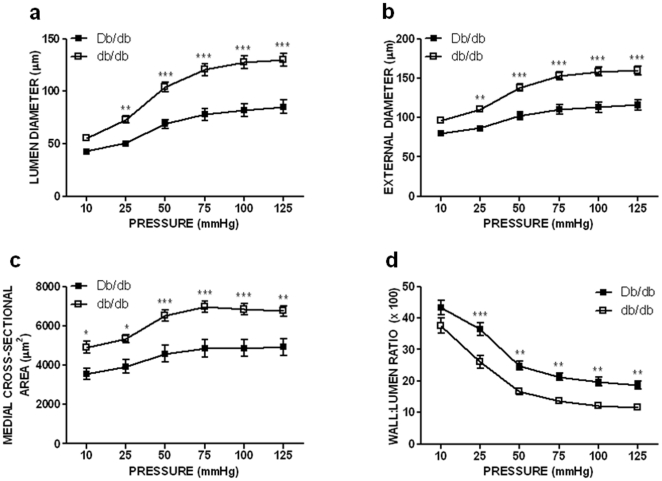
Lumen diameter (77.9±5.6 vs 120.6±5.5 µm at 75 mmHg, *p*<0.001), external diameter (110.4±6.4 vs 153.1±5.2 µm at 75 mmHg, *p*<0.001) and media cross-sectional area (4860.4±464.7 vs 6972.4±272.1 µm^2^ at 75 mmHg, *p*<0.001) were increased in 16 wk-old db/db MRAs while wall:lumen ratio (21.3±1.2 vs 13.7±0.8 at 75 mmHg, *p*<0.01) was decreased when compared to Db/db mice under physiological pressures of 50, 75 and 100 mmHg: a. Lumen diameter, b. external diameter, c. cross-sectional area, d. wall:lumen ratio. Two-way ANOVA: Db/db vs db/db, * p<0.05 ** p<0.01 *** p<0.001, means ± SEM; n = 8 for each group.

MRAs from 16 wk-old db/db mice had larger internal lumen diameter and external diameter measured at each pressure compared to MRAs isolated from control mice (Fig. 1a,b). Media cross-sectional area was also significantly increased in db/db mice compared to control mice (Fig. 1c); however, wall thickness was not different between groups (data not shown). As a result, the wall-to-lumen ratio was significantly decreased in db/db compared to control mice (Fig. 1d).

**Figure 2:**
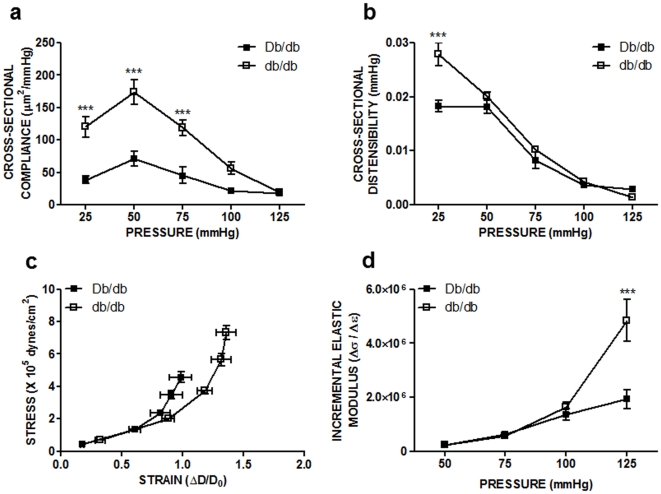
Compliance (45.1±12.7 vs 118.9±11.9 µm^2^/mmHg at 75 mmHg, *p*<0.001) was increased in db/db MRAs while cross-sectional distensibility (0.009±0.002 vs 0.010±0.0 kPa at 75 mmHg, *NS*) was unchanged when compared to Db/db mice under physiological pressures. The db/db stress-strain curve was shifted to the right of the Db/db MRA stress-strain curve, while incremental elastic modulus was mostly unchanged over a range of pressures, although an increase was observed at 125 mmHg: **a.** cross-sectional compliance, **b.** cross-sectional distensibility, **c.** stress-strain relationship, **d.** incremental elastic modulus. Two-way ANOVA: Db/db vs db/db,*** p<0.001, means ± SEM; n = 8 for each group.

MRAs from db/db mice were more compliant at low pressures than control mice (Fig. 2a). Neither the diabetic nor control mesenteric arteries displayed significant alterations in cross-sectional distensibility, except for a minor increase at 25 mmHg of pressure (Fig. 2b). Circumferential wall stress was significantly greater in db/db mesenteric arteries compared with control arteries at 75, 100, and 125 mmHg (data not shown). Finally, there was a rightward shift in the stress-strain relationship in db/db MRAs compared to control (Fig. 2c). Incremental elastic modulus, calculated from the incremental slopes of the stress-strain curve, was generally not different between normal and diabetic MRAs, although the diabetic MRAs were stiffer at the highest pressure (125 mmHg, Fig. 2d).

### Expression of Matrix Regulatory Proteins in Mesenteric Resistance Arteries in 16 wk-old Mice

**Figure 3:**
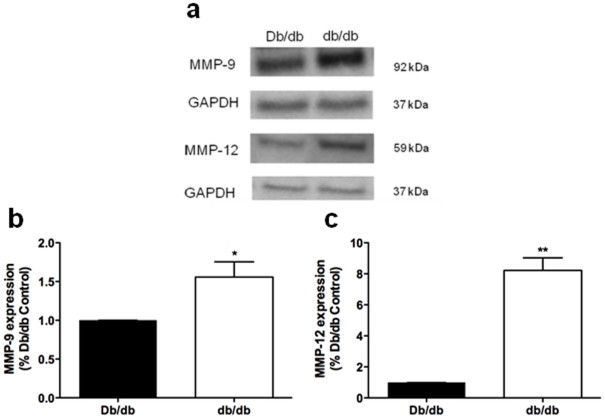
Increased vascular expression of and MMP-9 and MMP-12 in MRAs from diabetic db/db mice. **a.** Representative immunoblots of MMP-9, MMP-12 and GAPDH expression. **b.** Quantification of MMP-9, MMP-12 and GAPDH protein levels in Db/db and db/db mice. Unpaired t-test: Db/db vs db/db, *p<0.05, **p<0.005, means ± SEM; n = 3 tissue lysates pooled from 12 mice for each group.

Western blot analysis was used to determine the relationship between pro- and anti-fibrotic regulatory protein expression and ECM accumulation. MMP-9 and MMP-12 protein expression were significantly increased in MRAs isolated from diabetic db/db mice compared with the controls (Fig. 3a,b,c).

**Figure 4:**
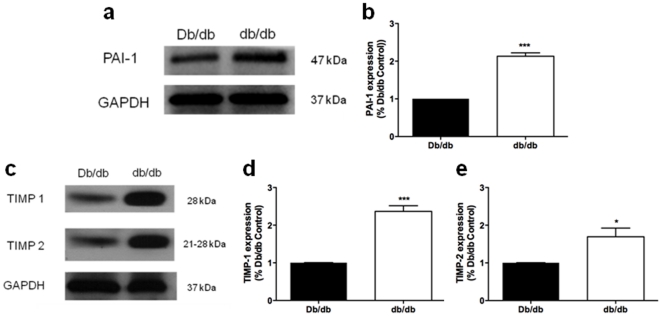
Increased vascular expression of and PAI-1, TIMP-1 and TIMP-2 in MRAs from diabetic db/db mice. **a.** Representative immunoblot of PAI-1 and GAPDH expression. **b.** Quantification of PAI-1 protein levels in Db/db and db/db mice. **c.** Representative immunoblot of TIMP-1, TIMP-2 and GAPDH expression. **d.** Quantification of TIMP-1, TIMP-2 and GAPDH protein levels in Db/db and db/db mice. Unpaired t-test: Db/db vs db/db, * p<0.05, *** p<0.001, means ± SEM; n = 3 tissue lysates pooled from 12 mice for each group.

**Figure 5:**
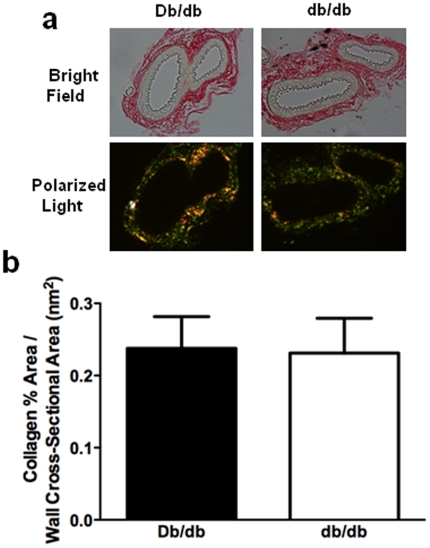
Vascular wall collagen content was unchanged in Db/db and db/db mice. **a.** Representative MRAs from Db/db and db/db mice stained with picrosirius red under both bright field and polarized light. **b.** Quantification of vascular wall collagen and normalized to wall cross-sectional area. Unpaired t-test: Db/db vs db/db, NS, means ± SEM; n = 8 for each group. Scale bars = 40 µm.

The expression of PAI-1 (Fig. 4a,b), and TIMP-1 and TIMP-2 (Fig. 4c,d,e), were significantly increased in db/db MRAs compared with control vessels. PSR staining indicated that these dynamic changes in pro- and anti-fibrotic protein expression did not result in a net change in collagen accumulation in db/db MRAs within the vascular wall (Fig. 5a,b).

### MRA remodeling and Superior Mesenteric Blood Flow in 12 wk-old Mice

**Figure 6:**
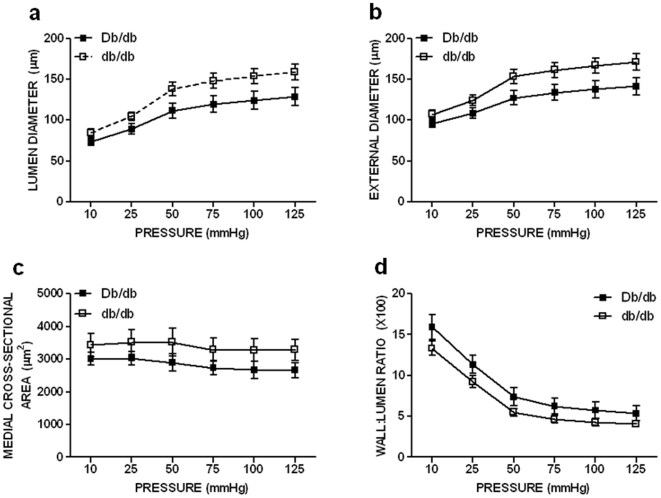
Lumen diameter (119.7±10.3 vs 147.8±9.2 µm at 75 mmHg, *p*>0.05), external diameter (133.7±9.7 vs 161.3±9.3 µm at 75 mmHg, *p*>0.05), media cross-sectional area (2736.3±224.1 vs 3289.1±360.1 µm^2^ at 75 mmHg, *p*>0.05), and wall:lumen ratio (6.2±1.0 vs 4.6±0.5 at 75 mmHg, *p*>0.05) of 12 wk-old diabetic MRAs were unchanged when compared to Db/db mice over a range of pressures: a. Lumen diameter, b. external diameter, c. medial cross-sectional area, d. wall:lumen ratio. Two-way ANOVA: Db/db vs db/db, NS, means ± SEM; n = 6 for each group.

**Figure 7:**
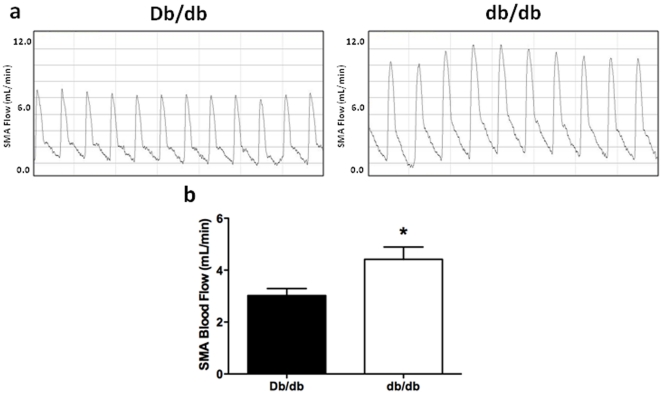
Superior mesenteric blood flow was increased in 12 wk-old diabetic db/db mice (Db/db 3.02±0.27 vs db/db 4.42±0.27 ml min^−1^; *p*<0.05). **a.** Representative tracings from actual blood flow recordings. **b.** Quantification of SMA blood flow. Unpaired t-test: Db/db vs db/db, * p<0.05, means ± SEM; n = 5 for Db/db and n = 6 for db/db.

We next determined whether changes in superior mesenteric blood flow preceded the observed hypertrophic outward remodeling in 16 wk-old db/db mice. At 12 wks of age, there was no observed MRA structural remodeling in db/db mice as evidenced by no differences in lumen diameter, external diameter, medial cross-sectional area, and wall:lumen ratio (Fig. 6a-d). MRA mechanics between normal and diabetic mice were also unchanged (data not shown). Conversely, superior mesenteric blood flow in db/db mice was significantly increased by 46% when compared with control mice (Fig. 7).

## Discussion

This study shows for the first time that MRAs in 16 wk-old type 2 DM db/db mice undergo hypertrophic outward remodeling associated with increased compliance at lower pressures and augmented stiffness at higher pressures. This remodeling process was accompanied by dynamic changes in the expression of MMP-9 and MMP-12, TIMP-1 and TIMP-2, and PAI-1 but no net change in medial collagen. These structural and biochemical findings were preceded by a 46% increase in superior mesenteric artery blood flow in 12 wk-old diabetic mice – a time point at which no MRA remodeling was present – suggesting that increased hemodynamic forces may precede and chronically influence downstream resistance artery remodeling.

Structural alterations of small arterial walls are the most potent predictors of adverse cardiovascular events in high-risk populations, including type 2 diabetic patients [23]. Arterial wall hypertrophy is present in resistance arteries (from gluteal biopsies) from patients with type 2 DM, with little to no change in intraluminal diameter [24]. Other studies in MRAs from streptozotocin-treated rats demonstrated either vascular hypertrophy [25] or eutrophic (i.e. no change in medial cross sectional area) outward remodeling as little as 3 weeks after experimentally-induced DM [26]. In contrast, our data reveal that MRAs in type 2 DM db/db mice had increased luminal and external diameters and medial cross-sectional areas, indicating hypertrophic outward remodeling. These structural changes were blood pressure-independent since we saw no significant differences in systolic blood pressure between groups (Table 1).

The reasons for these disparate remodeling processes are unclear but may reflect differences in vascular beds, insulin sensitivity, obesity and/or chronic exposure to hyperglycemia. Previous studies have shown that chronic changes in blood flow are one of the major stimuli dictating arterial diameter and reorganization of the vascular wall [27]. One possible explanation for our observations is that flow-induced hemodynamic changes may supersede the local neurohormonal and metabolic milieu that favor inward remodeling. Chronic hyperphagia in this model causes increased blood flow to accommodate increased nutrient uptake [28]. The thickness, composition and architecture of the artery wall are determined in part by stresses imposed by pressure and flow [29]. Both chronic increases in blood flow and wall stress trigger a combined response of hypertrophic and outward remodeling [18]. As shown by us and others, blood flow to the small intestine is also increased in the diabetic animals secondary to prolonged hyperphagia and may act as a stimulus for outward remodeling [30]. This is consistent with data showing outward hypertrophic remodeling of resistance arteries is the result of a chronic increase in blood flow [18], [31], [32], [33]. Our data support the notion that increased blood flow resulting from prolonged feeding in obese db/db mice outweigh the influences of hyperglycemia, inflammation, and oxidative stress that dictate vessel wall remodeling in DM. Given that each vascular bed is exposed to a unique pattern of hemodynamic, neurohormonal and inflammatory stimuli, the effects of type 2 DM on vascular remodeling cannot be globally applied to all resistance beds.

Vessel wall remodeling also leads to changes in the structural mechanical properties. Arterial compliance, defined as the change in lumen area for a given change in pressure, depends on the size of the vascular lumen as a hollow structure and cushions changes in arterial pulse pressure. Distensibility represents compliance corrected for vascular lumen size and depends on the medial mass and intrinsic elastic properties of the vascular wall [26], [34]. E_inc_ reflects the mechanical properties of the vascular wall material (collagen, vascular smooth muscle, elastin and other ECM components) independent of geometry. Our results show increased MRA cross-sectional compliance in db/db mice, resulting in a less stiff vessel at lower physiological pressures. Conversely, there were no differences in cross-sectional distensibility between normal and diabetic mice, except for an increase at 25 mmHg. These data are consistent with previous reports that increased arterial compliance fails to correlate with an increase in arterial distensibility [26], [34], which are likely due to the combination of increased lumen diameter and wall stress without alterations in wall thickness. The stress–strain relationship is an additional geometry-dependent measure of vascular compliance. In general, leftward deviations in this parameter functionally describe a stiffer, less compliant vessel [35]. In contrast, our results show a rightward deviation in the stress-strain curve in diabetic MRAs, indicating a less stiff, more compliant vessel compared to non-diabetic controls in the face of an unchanged incremental modulus, except for a transient increase at higher physiological pressure. These data are the first to report increased MRA cross-sectional compliance in type 2 DM mice, since most studies have reported either a leftward shift or no differences in the stress-strain relationship [36], [37]. The current study also demonstrates that as intraluminal pressure increases, wall stress increases in diabetic mice compared to controls, which is a function of and directly proportional to increased luminal diameter. Previous studies have shown that changes in wall stress result in either impaired myogenic response (stimulus for vascular hypertrophy) or in altered vessel geometry to reduce wall stress [14], [18], [24], [38], [39].

The molecular mechanisms that govern ECM remodeling in diabetic resistance arteries have not been extensively studied. Here we show that type 2 DM MRA structural remodeling was accompanied by no net matrix accumulation. This was not surprising, since matrix accumulation is usually associated with vessel stiffness and inward remodeling [40]. However, the concomitant increase in both anti-fibrotic (MMP-9 and MMP-12) and pro-fibrotic (TIMP-1, TIMP-2 and PAI-1) molecules is indicative of dynamic ECM turnover. We speculate that the ECM is in a constant state of flux as the vessel undergoes outward remodeling in an attempt to maintain wall integrity. The mechanisms involved in the induction of matrix regulatory proteins are unclear but may reflect both hemodynamic and humoral responses. Increased MMP-9 and MMP-12, which can hydrolyze elastin, may be secondary to reactive oxidative species and inflammation in diabetic vessels. Uemura et al. demonstrated an increase in MMP-9 activity, mRNA and protein expression in endothelial cells exposed to hyperglycemia, which may facilitate the pro-inflammatory response in the vessel wall [41]. On the other hand, the increased expression of pro-fibrotic factors, such as PAI-1 and ECM accumulation is consistent with DM-induced nephropathy and vasculopathy [17], [42]. Similarly, other groups report an imbalance between MMP and TIMP levels that lead to increased synthesis and accumulation of matrix proteins in the heart and kidneys of experimental models of DM [15], [16]. Plasma and urine samples from human diabetic patients show marked increases in MMP and TIMP concentrations, reflecting abnormal ECM turnover [43], [44]. It is also important to note that it is the overall organization of the ECM into a 3-dimensional network, as well as the fluid properties of the non-collagenous components of the ECM (elastin, fibrin) that will ultimately impart elasticity and compliance to the tissue [45]. Thus the overall balance between anti-fibrotic and pro-fibrotic factors at this time point maintains ECM integrity without accumulation in an attempt limit outward vessel remodeling. Future studies examining the temporal and spatial profile of MMPs and TIMPs in DM-induced microvascular remodeling are warranted.

In summary, our study shows significant hypertrophic outward remodeling of MRAs from obese, type 2 diabetic db/db mice that is accompanied by increased compliance and wall stress. This remodeling likely reflects an integrated response of a combination of increased regional hemodynamics that precedes structural adaptation, type 2 DM-dependent insulin resistance, inflammation, obesity, and oxidative stress. Changes in the balance between pro- and anti-fibrotic factors resulted in no net ECM accumulation that work in concert to reduce wall stress and limit outward remodeling.

## References

[1] (2008). Economic costs of diabetes in the U.S. In 2007 Diabetes Care.

[2] Dhalla NS, Liu X, Panagia V, Takeda N (1998). Subcellular remodeling and heart dysfunction in chronic diabetes.. Cardiovasc Res.

[3] Laakso M (1999). Hyperglycemia and cardiovascular disease in type 2 diabetes.. Diabetes.

[4] Cooper ME (2004). The role of the renin-angiotensin-aldosterone system in diabetes and its vascular complications.. Am J Hypertens.

[5] Goh SY, Cooper ME (2008). Clinical review: The role of advanced glycation end products in progression and complications of diabetes.. J Clin Endocrinol Metab.

[6] Stockklauser-Farber K, Ballhausen T, Laufer A, Rosen P (2000). Influence of diabetes on cardiac nitric oxide synthase expression and activity.. Biochim Biophys Acta.

[7] Ding H, Howarth AG, Pannirselvam M, Anderson TJ, Severson DL (2005). Endothelial dysfunction in Type 2 diabetes correlates with deregulated expression of the tail-anchored membrane protein SLMAP.. Am J Physiol Heart Circ Physiol.

[8] Lagaud GJ, Masih-Khan E, Kai S, van Breemen C, Dube GP (2001). Influence of type II diabetes on arterial tone and endothelial function in murine mesenteric resistance arteries. J Vasc Res.

[9] Heagerty AM, Aalkjaer C, Bund SJ, Korsgaard N, Mulvany MJ (1993). Small artery structure in hypertension.. Dual processes of remodeling and growth Hypertension.

[10] Mulvany MJ, Baumbach GL, Aalkjaer C, Heagerty AM, Korsgaard N (1996). Vascular remodeling.. Hypertension.

[11] Rizzoni D, Porteri E, Guelfi D, Muiesan ML, Piccoli A (2001). Endothelial dysfunction in small resistance arteries of patients with non-insulin-dependent diabetes mellitus.. J Hypertens.

[12] Rizzoni D, Porteri E, Guelfi D, Muiesan ML, Valentini U (2001). Structural alterations in subcutaneous small arteries of normotensive and hypertensive patients with non-insulin-dependent diabetes mellitus.. Circulation.

[13] Brassard P, Amiri F, Schiffrin EL (2005). Combined angiotensin II type 1 and type 2 receptor blockade on vascular remodeling and matrix metalloproteinases in resistance arteries.. Hypertension.

[14] Briones AM, Gonzalez JM, Somoza B, Giraldo J, Daly CJ (2003). Role of elastin in spontaneously hypertensive rat small mesenteric artery remodelling.. J Physiol.

[15] Han SY, Jee YH, Han KH, Kang YS, Kim HK (2006). An imbalance between matrix metalloproteinase-2 and tissue inhibitor of matrix metalloproteinase-2 contributes to the development of early diabetic nephropathy.. Nephrol Dial Transplant.

[16] Li Q, Sun SZ, Wang Y, Tian YJ, Liu MH (2007). The roles of MMP-2/TIMP-2 in extracellular matrix remodelling in the hearts of STZ-induced diabetic rats.. Acta Cardiol.

[17] McFarlane SI, Kumar A, Sowers JR (2003). Mechanisms by which angiotensin-converting enzyme inhibitors prevent diabetes and cardiovascular disease.. Am J Cardiol.

[18] Pourageaud F, De Mey JG (1997). Structural properties of rat mesenteric small arteries after 4-wk exposure to elevated or reduced blood flow.. Am J Physiol.

[19] Gibbons GH, Dzau VJ (1994). The emerging concept of vascular remodeling.. N Engl J Med.

[20] Baumbach GL, Heistad DD (1989). Remodeling of cerebral arterioles in chronic hypertension.. Hypertension.

[21] Schiffrin EL (1992). Reactivity of small blood vessels in hypertension: relation with structural changes.. State of the art lecture Hypertension.

[22] Stewart JA, Wei CC, Brower GL, Rynders PE, Hankes GH (2003). Cardiac mast cell- and chymase-mediated matrix metalloproteinase activity and left ventricular remodeling in mitral regurgitation in the dog.. J Mol Cell Cardiol.

[23] Rizzoni D, Porteri E, Boari GE, De Ciuceis C, Sleiman I (2003). Prognostic significance of small-artery structure in hypertension.. Circulation.

[24] Schofield I, Malik R, Izzard A, Austin C, Heagerty A (2002). Vascular structural and functional changes in type 2 diabetes mellitus: evidence for the roles of abnormal myogenic responsiveness and dyslipidemia.. Circulation.

[25] Cooper ME, Rumble J, Komers R, Du HC, Jandeleit K (1994). Diabetes-associated mesenteric vascular hypertrophy is attenuated by angiotensin-converting enzyme inhibition.. Diabetes.

[26] Crijns FR, Wolffenbuttel BH, De Mey JG, Struijker Boudier HA (1999). Mechanical properties of mesenteric arteries in diabetic rats: consequences of outward remodeling.. Am J Physiol.

[27] Langille BL, Bendeck MP, Keeley FW (1989). Adaptations of carotid arteries of young and mature rabbits to reduced carotid blood flow.. Am J Physiol.

[28] Cox JE, Powley TL (1977). Development of obesity in diabetic mice pair-fed with lean siblings.. J Comp Physiol Psychol.

[29] Glagov S, Vito R, Giddens DP, Zarins CK (1992). Micro-architecture and composition of artery walls: relationship to location, diameter and the distribution of mechanical stress.. J Hypertens.

[30] Friedman MI, Ramirez I (1994). Food intake in diabetic rats: relationship to metabolic effects of insulin treatment.. Physiol Behav.

[31] Unthank JL, Fath SW, Burkhart HM, Miller SC, Dalsing MC (1996). Wall remodeling during luminal expansion of mesenteric arterial collaterals in the rat.. Circ Res.

[32] Tuttle JL, Nachreiner RD, Bhuller AS, Condict KW, Connors BA (2001). Shear level influences resistance artery remodeling: wall dimensions, cell density, and eNOS expression.. Am J Physiol Heart Circ Physiol.

[33] Tulis DA, Unthank JL, Prewitt RL (1998). Flow-induced arterial remodeling in rat mesenteric vasculature.. Am J Physiol.

[34] Van Bortel LM, Kool MJ, Boudier HA, Struijker Boudier HA (1995). Effects of antihypertensive agents on local arterial distensibility and compliance.. Hypertension.

[35] Dunn WR, Gardiner SM (1997). Differential alteration in vascular structure of resistance arteries isolated from the cerebral and mesenteric vascular beds of transgenic [(mRen-2)27], hypertensive rats.. Hypertension.

[36] Briones AM, Salaices M, Vila E (2007). Mechanisms underlying hypertrophic remodeling and increased stiffness of mesenteric resistance arteries from aged rats.. J Gerontol A Biol Sci Med Sci.

[37] Wigg SJ, Tare M, Forbes J, Cooper ME, Thomas MC (2004). Early vitamin E supplementation attenuates diabetes-associated vascular dysfunction and the rise in protein kinase C-beta in mesenteric artery and ameliorates wall stiffness in femoral artery of Wistar rats.. Diabetologia.

[38] Qiu HY, Valtier B, Struyker-Boudier HA, Levy BI (1995). Mechanical and contractile properties of in situ localized mesenteric arteries in normotensive and spontaneously hypertensive rats.. J Pharmacol Toxicol Methods.

[39] Intengan HD, Thibault G, Li JS, Schiffrin EL (1999). Resistance artery mechanics, structure, and extracellular components in spontaneously hypertensive rats : effects of angiotensin receptor antagonism and converting enzyme inhibition.. Circulation.

[40] Arribas SM, Hinek A, Gonzalez MC (2006). Elastic fibres and vascular structure in hypertension. Pharmacol Ther.

[41] Uemura S, Matsushita H, Li W, Glassford AJ, Asagami T (2001). Diabetes mellitus enhances vascular matrix metalloproteinase activity: role of oxidative stress. Circ Res.

[42] Wang W, Koka V, Lan HY (2005). Transforming growth factor-beta and Smad signalling in kidney diseases.

[43] Derosa G, D'Angelo A, Tinelli C, Devangelio E, Consoli A (2007). Evaluation of metalloproteinase 2 and 9 levels and their inhibitors in diabetic and healthy subjects.. Diabetes Metab.

[44] Thrailkill KM, Bunn RC, Moreau CS, Cockrell GE, Simpson PM (2007). Matrix metalloproteinase-2 dysregulation in type 1 diabetes.. Diabetes Care.

[45] Goldsmith EC, Borg TK (2002). The dynamic interaction of the extracellular matrix in cardiac remodeling.. J Card Fail.

